# Application of Adaptive Evolution to Improve the Stability of Bacteriophages during Storage

**DOI:** 10.3390/v12040423

**Published:** 2020-04-09

**Authors:** Kelvin K. Kering, Xiaoxu Zhang, Raphael Nyaruaba, Junping Yu, Hongping Wei

**Affiliations:** 1 Key Laboratory of Special Pathogens and Biosafety, Wuhan Institute of Virology, Center for Biosafety Mega-Science, Chinese Academy of Sciences, Wuhan 430071, China; keringk@gmail.com (K.K.K.); zxxwhiov@163.com (X.Z.); rohuru1@gmail.com (R.N.); yujp@wh.iov.cn (J.Y.); 2 University of Chinese Academy of Sciences, Beijing 100049, China; 3 Sino-Africa Joint Research Center, Nairobi 00200, Kenya

**Keywords:** bacteriophage, phage therapy, biocontrol, adaptation, natural selection, thermal stability, genetic variation

## Abstract

Phage stability is important for the successful application of bacteriophages as alternative antibacterial agents. Considering that temperature is a critical factor in phage stability, this study aimed to explore the possibility of improving long-term phage stability through adaptive evolution to elevated temperature. Evolution of three wild-type ancestral phages (*Myoviridae* phage Wc4 and *Podoviridae* phages CX5 and P-PSG-11) was induced by subjecting the phages to heat treatment at 60 °C for five cycles. The adapted phages showed better stability than the wild-type ancestral phages when subjected to heat treatment at 60 °C for 1 h and after 60 days of storage at 37 °C. However, the adapted phages could not withstand thermal treatment at 70 °C for 1 h. The infectivity and the lytic properties of the phages were not changed by the evolution process. Whole-genome sequencing revealed that single substitutions in the tail tubular proteins were the only changes observed in the genomes of the adapted phages. This study demonstrates that adaptive evolution could be used as a general method for enhancing the thermal stability of phages without affecting their lytic activity. Sequencing results showed that bacteriophages may exist as a population with minor heterogeneous mutants, which might be important to understand the ecology of phages in different environments.

## 1. Introduction

Bacteriophages (phages) are viruses that replicate within bacteria, often resulting in the lysis of the bacterial cell. With an estimated total world-wide number of 10^31^, phages are considered the most abundant forms of life on earth [1,2,3]. They are present in every environment where bacteria can be found: animals, humans, plants, water, soil, and sewage [4,5,6,7]. Research on phage ecology has also shown that desert soil (Sahara and Namib) contains abundant and diverse phage populations [8,9]. These desert soil phage populations vary considerably from those in aquatic systems, since they can withstand intense UV radiation and high temperatures [10]. Due to the increase in antimicrobial resistance, there also has been an upsurge in studies of phages as plausible antibacterial agents in clinical medicine, agriculture, food processing, and surface decontamination [11,12,13,14]. The interest in phages as antibacterial agents is attributed to their specificity, self-replication, minimum disruption of normal flora, low inherent toxicity, biofilm clearance, and counteractive adaptation against resistant bacteria [15].

Phage stability is a critical aspect for ensuring the activity of phages during transportation and storage after preparation [16]. Considering that phages are primarily made of a protein envelope encapsulating nucleic acid, most of the strategies exploited in stabilizing phages are similar to those applied to protein-based therapeutics [17]. The conventional strategies of stabilizing liquid phage preparations involve adding excipients (e.g., sugars and polyols) into the phage preparations and/or converting the liquid formulations into powder [16]. Powder formulations are widely considered to be more stable than liquid formulations [18]. Liquid preparations can be converted into powder formulations through lyophilization/freeze drying [17,19], spray drying [18,20], and spray freeze-drying [21]. However, besides the extra cost, phage activity could be reduced during this process, and different settings would be needed for converting liquid preparations of phages into powder. 

Temperature is a critical factor in the survivability of phages as it influences the occurrence, activity, and storage of phages [22]. It is therefore worthy to explore the effect of adaptive thermal evolution on the stability of phages during storage. Adaptive evolution has previously been used to adapt phages to different environmental conditions such as various temperatures [23,24,25,26,27] and ultraviolet (UV) light [28]. Despite the knowledge acquired from previous evolutionary adaptation studies of phages, there is a paucity of information on whether evolving phages with respect to an elevated temperature has an impact on the stability of phages during storage at a lower temperature.

The phytobacterial pathogens *Ralstonia solanaceraum* and *Pectobacterium* species are a major hindrance to the global production of potatoes. *R. solanacerum* causes bacterial wilt in the plant while in the field and brown rot in the tuber [29]. *Pectobacterium carotovorum* subsp. *carotovorum* (Pcc) and *Pectobacterium atrosepticum* (Pba) cause blackleg in the growing plant and soft rot in the potato tuber during storage and transportation [30]. Phages have been considered in the biocontrol of these phytopathogens [31,32]. A major challenge in phage-mediated biocontrol of phytopathogens is phage inactivation by UV light, desiccation, and excess heat. It is therefore essential to ensure that phages can remain active during storage and following application in a plant environment. 

Since most phage preparations are frequently utilized in an aqueous form [19], it is interesting to explore the possibility of maintaining or enhancing phage stability during storage in ambient conditions or elevated temperatures without the use of excipients and/or conversion of liquid formulations to powder. In this study, we aimed to adapt phages to an elevated temperature, compare the stability of the ancestral phages and the adapted phages during storage, and identify any genomic changes in the adapted phages.

## 2. Materials and Methods 

### 2.1. Bacterial Strains, Phages, and Culture Conditions

The bacterial hosts and phages used in this study are listed in Table 1.

The bacterial strains Pcc KPM17 and Pba WHG10001 were routinely cultured on Luria–Bertani agar plates (1.5% *wt*/*vol*, agar) and broth at 28 °C while *R. solanaceraum* GIM1.74 was cultured on CTG agar plates (1% casamino acid hydrosylate, 1% tryptone, and 1.5% *wt*/*vol*, agar) and broth at 28 °C. Tris-HCl buffer pH 7.5 (50 mM Tris-base, 150 mM NaCl, 10 mM MgCl_2_·6H_2_O, and 2 mM CaCl_2_) was used for routine phage experiments. 

### 2.2. Phage Adaptive Evolution

To determine the thermal stability of the phages, 500 µL of the lysates at concentrations of ca. 10^9^ PFU/mL was subjected to heat treatment at 50 °C, 60 °C, and 70 °C in a water bath for 1 h. The temperature that resulted in a substantial decline of phage titer without complete loss of activity for all the three phages was considered for downstream experimental evolution processes. 

The phages were evolved as previously described [26] with a few modifications. Briefly, 500 µL of phage lysate in 1.5 mL Eppendorf tubes was subjected to heat treatment at 60 °C in a water bath for 1 h. The surviving phages were then allowed to cool to room temperature and amplified by mixing an equal volume (500 µL) of the phage lysate and its respective host solution and then incubating at 28 °C, 160 rpm for 15 min to allow phage–host adsorption. The lysate was then added to 4 mL of soft agar, poured as an overlay onto agar plates, and incubated overnight at 28 °C. The surviving phages (now as plaques) were obtained by scraping off the overlay on agar plates into a 15 mL centrifuge tube containing 2 mL Tris-HCl buffer, which was vortexed and centrifuged at 5500 rpm for 15 min at 4 °C. Five hundred microliters of the phage lysate obtained in the supernatant was then transferred to 1.5 mL Eppendorf tubes, subjected to heat treatment again at 60 °C for 1 h, and then amplified as explained above. This process was repeated for five cycles to obtain heat-adapted phages. 

### 2.3. Stability of the Ancestral and Heat-Adapted Phages 

The ancestral phages and the adapted phages were subjected to heat treatment in a water bath at 60 °C for 1 h to evaluate the short-term stability of the phages. The stability of the phages was also evaluated after 14, 30, and 60 days during storage at 37 °C. Reduction in phage titer was used to compare the difference in stability between the ancestral phages and the adapted phages. Phage titers were determined by diluting the phages in 10-fold serial dilutions in Tris-HCl buffer, and 10 µL of the dilutions was spotted on the double agar overlay containing their respective host bacteria, allowed to adsorb, and incubated overnight at 28 °C.

### 2.4. Determination of the Host Range and Lytic Curve Pattern of the Phages 

To evaluate whether there was a trade-off between stability and lytic efficacy in the adapted phages, the host range and the lytic curve patterns of the ancestral phages and the adapted phages were compared. Host range analysis was performed as previously described [33]. Briefly, an overnight bacterial culture (500 μL) was mixed with 4 mL of soft agar and poured on agar plates. Ten microliters of serially diluted phage solution was then spotted on the respective double agar overlay, allowed to adsorb, and incubated overnight at 28 °C. The presence of clear zones and plaques on the spotted section of the lawn was used as an indication of the ability to lyse the bacteria. The lytic curve pattern was determined by measuring the change in bacterial optical density (OD_600_) during a 12 h incubation. The liquid medium (100 μL) was transferred into a 96-well culture plate first. The overnight host–bacterial (50 μL) culture (10^8^ CFU/mL) was then added and mixed, followed by adding the respective phage lysate (50 μL). For control experiments, the liquid medium was mixed with the bacteria or phage and double-distilled water. The changes in OD_600_ of the final mixtures were then monitored continuously using a microplate reader (SynergyH1, BioTek, Winooski, VT, USA) for 12 h at 28 °C. The experiment was repeated three times.

### 2.5. Genome Extraction, Sequencing, and Analysis

A single plaque of the ancestral and adapted phages was picked and propagated by double agar overlays to obtain 40 mL of phage lysate for DNA extraction. Solid sodium chloride and PEG 8000 were added to the lysate to concentrations of 1 M and 10% (*w*/*v*) respectively. This was dissolved and incubated at 4 °C overnight. The phage lysate was centrifuged at 10,000 rpm for 20 min at 4 °C. The pellet obtained was re-suspended in 500 µL of Tris-HCl buffer and transferred to a clean 2 mL Eppendorf tube. Exogenous nucleic acids were removed from the phage lysates by treatment with 2.5 µl of DNase I (3 U/µL, RNase free, Thermo Fisher Scientific) and 1.5 µL of RNase (20 mg/mL, Thermo Fisher Scientific). The lysates were then treated with 0.2 M EDTA, proteinase K, and 10% SDS. Extraction of phage genomes was done through the phenol–chloroform protocol as described previously [34]. The quality and quantity of the DNA were determined using a Nanodrop reader (ND-2000, Thermo Fisher, Waltham, MA, USA). 

Genome sequencing was done by Sangon Biotech (Shanghai, China) using an Illumina HiSeq sequencer. The sequencing was paired-end of 150 bp ×2. The reads were trimmed using Trimmomatic-0.36 [35], and quality assessment was done using FastQC (v 0.11.8) to remove adaptors and reads with low-quality bases. De novo assembly was done using SPAdes 3.11.1 [36] or MEGAHIT [37] with default settings. The whole genome sequences of phages were aligned with the NT database to identify potential homologues using Blastn [38]. Complete genomes sequences of 40 *Ralstonia* phages and 63 *Pectobacterium* phages were downloaded from the GenBank database to perform multiple sequence alignment with the genome sequences of phages in this study. The alignment was performed using MAFFT software with default settings [39]. The phylogenetic trees were constructed by FastTree software (Version: 2.1.4) [40] and visualized using the ITOL online website (https://itol.embl.de/). Annotation of complete genomes was performed using Prokka v 1.12 [41], and the coding sequences (CDS) were predicted using Prodigal [42]. The putative function of the mutated genes was predicted through alignment with the NR database using Blastp [38]. The genome sequences were deposited in GenBank (NCBI) under the accession numbers MN270887 (CX5), MN270888 (CX5-1), MN270889 (P-PSG-11), MN270890 (P-PSG-11-1), MN270891 (Wc4), and MN270892 (Wc4-1). 

Comparative genome analysis between the ancestral and the adapted phages was performed by two programs (NUCmer and show-snps) of Mummer 3.23 software package [43] to identify any changes in the genomic sequences of the adapted phages. NUCmer (NUCleotide MUMmer) is suitable for the alignment of highly similar nucleotide sequences, and show-snps is a program for reporting single-nucleotide polymorphisms (SNPs) contained in a delta file output by NUCmer. The SNPs between ancestral and adapted phages were identified by the two steps mentioned above. After this, alignment between 150 bp Illumina raw reads archived by high-throughput sequencing with reference genome sequences was done using Bwa software (Version: 0.7.17-r1188) [44]. In the alignment process, whole-genome sequences of the ancestral phage and adapted phages were used as references, and the number of nucleotides mapped to the corresponding position was calculated by Samtools software [45]. The sequences of the mutated genes were aligned with those of the ancestral phages using MEGA 7.0.26 [46]. The mutated genes were confirmed by PCR and sequenced using the ABI 3730 DNA analyzer (Applied Biosystems™). Virfam [47] was used to predict the morphology of the phages using the genomic sequences and confirmed by transmission electron microscopy. 

### 2.6. Electron Microscopy

The ancestral phages (Wc4, CX5 and P-PSG-11) were amplified and concentrated using a CsCl density gradient (2 mL of each of 1.45 g/mL, 1.50 g/mL, and 1.70 g/mL solutions of CsCl in Tris-HCl buffer) and ultracentrifugation at 35,000 rpm at 4 °C for 2 h. The phage band (blue) collected was dialyzed using a cellulose membrane in Tris-HCl buffer for 12 h. Then, 20 µL of phage suspensions was placed on a carbon-coated copper grid and allowed to adsorb for 10 min. Excess liquid was drawn off carefully using filter paper, and the grid was allowed to air-dry. Phages were negatively stained with freshly prepared 2% phosphotungstic acid (PTA) for 3 min. The copper grids were air-dried for 2 h and then observed under a transmission electron microscope (Instrument model: H-7000FA; Manufacturer: Hitachi Japan) at an operating voltage of 75 kv.

## 3. Results

### 3.1. Thermal Stability of the Phages

An initial thermal stability test was done to evaluate the temperature at which to evolve the phages. Thermal treatment of the ancestral phages (Wc4, CX5 and P-PSG-11) at 50 °C caused a slight titer decline of less than 1 log in all three phages. At 60 °C, there was a titer decline in all three phages, without complete loss of activity. However, at 70 °C, the three phages lost all the activity, as no plaques were observed on the double-layer agar plates. Based on these results, 60 °C was chosen as the temperature to evolve the phages. 

### 3.2. Phage Evolution 

After the first cycle of heating and amplification in the evolution process, a decline in the phage population was observed, since few plaque-forming units (PFU) were observed on the bacterial lawn. Pcc KPM17 phage Wc4 showed the most drastic decline of PFU on the bacterial lawn. However, as the number of cycles increased, the number of PFU on the bacterial lawn following the same treatment protocol also increased progressively. These phenomenon suggested that the phages were adapting to the thermal pressure applied. By the fifth cycle, the entire bacterial lawn could be found cleared by the phages, which was not previously the case, indicating a minimal or no reduction of the activity of the phages by heating. 

### 3.3. Stability of the Ancestral and Adapted Phages at 60 °C

**Figure 1:**
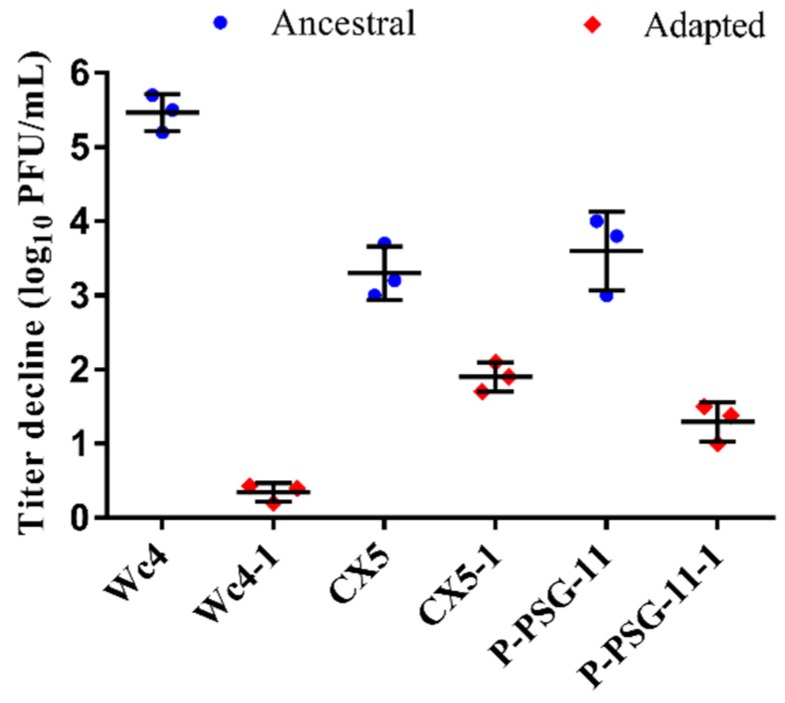
Titer reduction of the ancestral phages (blue) and the adapted phages (red) after heat treatment at 60 °C for 1 h. Error bars represent ± SD. (*n* = 3).

The stability of the adapted phages was compared to that of the ancestral phages at 60 °C and 70 °C for 1 h. The adapted phages showed better stability than the wild-type ancestral phages when subjected to heat treatment at 60 °C (Figure 1). Pcc KPMI7 phage Wc4 had a titer loss of 5.47 ± 0.25 log, while the adapted phage Wc4-1 showed a titer decline of 0.34 ± 0.13 log under the same conditions. Phage CX5 showed a titer reduction of 3.30 ± 0.36 log compared to the adapted phage CX5-1, which exhibited a decline of 1.90 ± 0.20 log. *R. solanaceraum* phage P-PSG-11 had a titer reduction of 3.60 ± 0.53 log, while the adapted phage P-PSG-11-1 showed a titer reduction of 1.29 ± 0.26 log. However, the adapted phages could still not withstand heat treatment at 70 °C for 1 h, as no plaques were observed on the bacterial lawn after heat treatment at this temperature.

### 3.4. Stability of the Ancestral and Adapted Phages at 37 °C 

**Figure 2:**
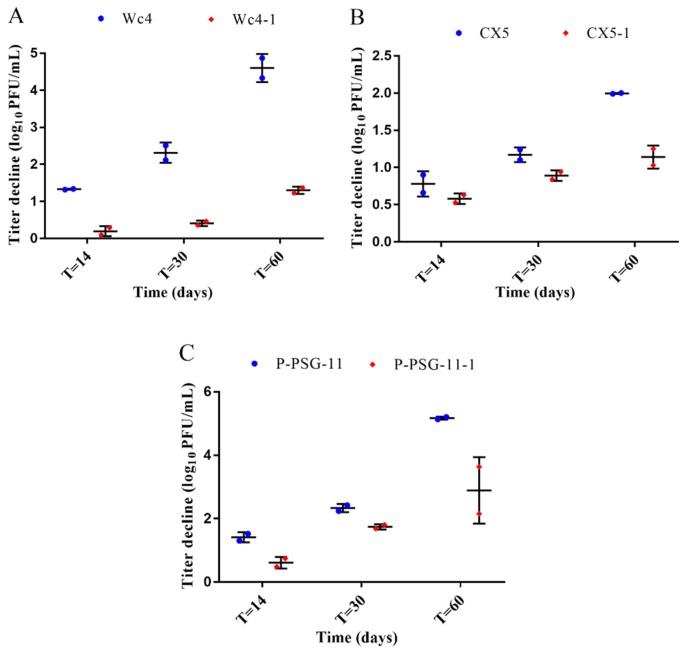
Titer reduction of the ancestral phages (blue) and the adapted phages (red) after 60 days of incubation at 37 °C. (**A**) Wc4 and Wc4-1 (**B**) CX5 and CX5-1 (**C**) P-PSG-11 and P-PSG-11-1. The results are the mean values of two independent experiments; error bars represent ± standard deviation of the phage titer obtained.

The stability of the ancestral and adapted phages was evaluated during 60 days of storage at 37 °C. The three ancestral phages showed higher titer declines compared to the adapted phages. After 60 days of storage, a titer loss of 4.6 ± 0.38 log was observed for the ancestral phage Wc4, while the titer reduction for the adapted phage Wc4-1 was 1.3 ± 0.099 log (Figure 2A). Phage CX5 showed a decline in titer of 1.995 ± 0.01 log, while titer decline for the adapted phage CX5-1 was 1.14 ± 0.16 log during the same period (Figure 2B). A titer reduction of 5.17 ± 0.05 log was observed for the ancestral phage P-PSG-11, while the adapted phage P-PSG-11-1 showed a reduction of 2.89 ± 1.05 log (Figure 2C). Although the phage titers were reduced with time for both the ancestral and the adapted phages during storage, the adapted phages showed better stability than the ancestral phages, indicating that the adaptive evolution process had an impact on the stability of the phages.

### 3.5. Determination of Infectivity and Lytic Curve Patterns 

To determine if there was a trade-off between stability and infectivity, the host range and lytic curve patterns of the adapted and ancestral phages were compared. As shown in Supplementary Figure S1, the lytic curve patterns of the ancestral phages and adapted phages were moderately similar throughout the phage infection and lysis of the bacteria. It was also observed that the adapted phages were able to lyse all the bacterial strains that were sensitive to the ancestral phages. Clear zones and plaques were observed on the sections of bacterial lawns of 16 Pcc strains and 3 *R. solanacearum* (Supplementary Tables S1 and S2) strains that were tested against their respective phages. The adapted phages CX5-1 and the ancestral phage CX5 showed similar lytic activity on *P. atrosecpticum* WHG10001.

### 3.6. Genomic Sequence Analysis

**Figure 3:**
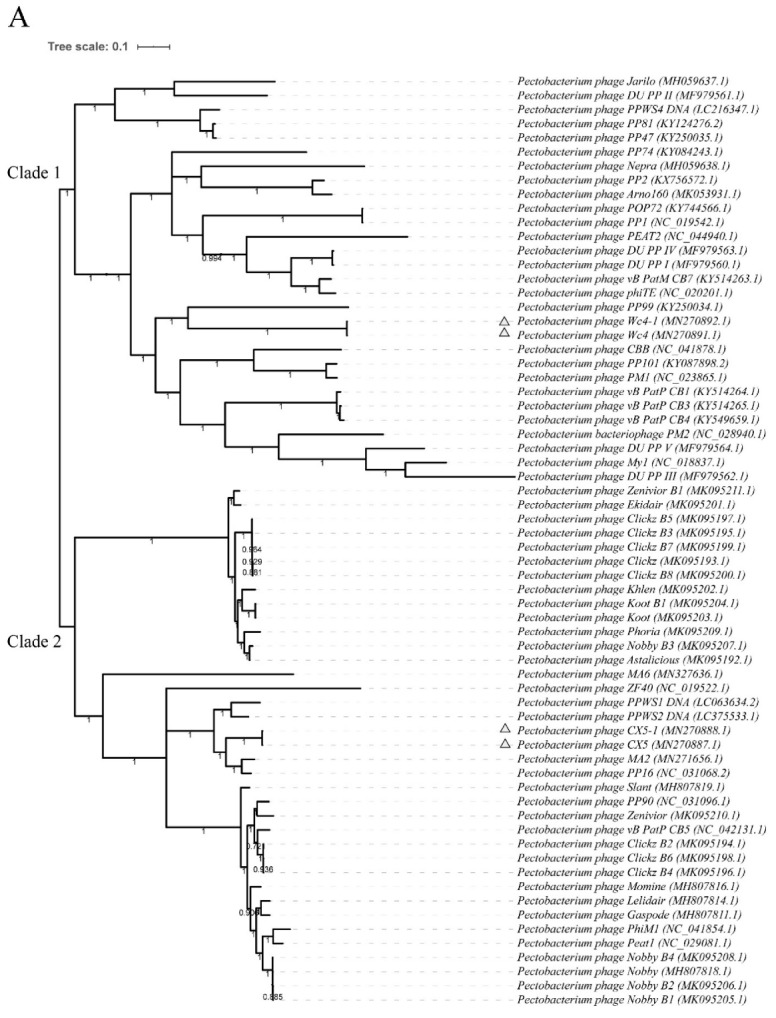

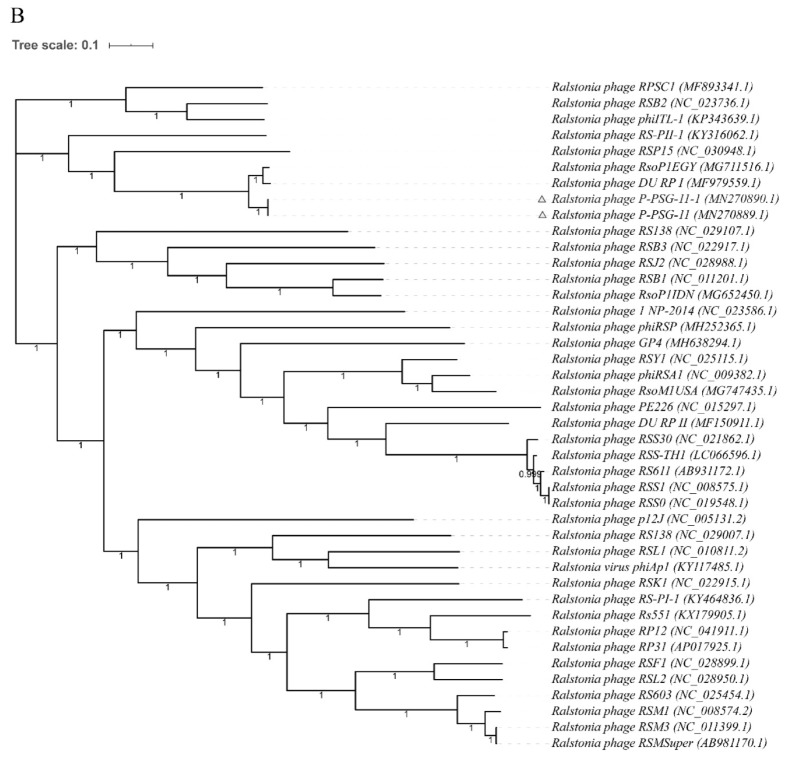
Phylogenetic analysis of the ancestral and adapted phages showing their relationship with closely related phages from the GenBank database. (**A**) Relationship of *Pectobacterium* phages (Wc4, Wc4-1, CX5, and CX5-1) with other 63 *Pectobacterium* phages. (**B**) Relationship of *Ralstonia* phages (P-PSG-11 and P-PSG-11-1) with other 40 *Ralstonia* phages. The trees were constructed using the maximum likelihood method based on the complete sequences of phages in 1000 bootstrap replications.

**Figure 4:**
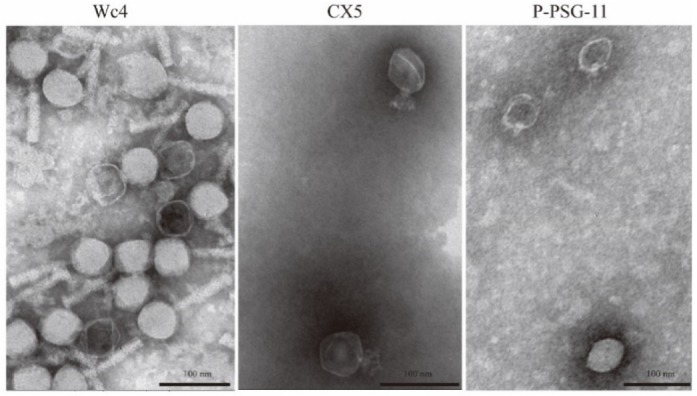
Transmission electron microscopy images of phage Wc4, CX5, and P-PSG-11. Scale bar, 100 nm.

Genome analysis revealed that the genome length of the adapted phages was consistent with that of the corresponding ancestral phage. Phages Wc4 and Wc4-1 contain 92,039 bp of DNA with 144 predicted CDS, phages CX5 and CX5-1 contain 43,885 bp of DNA with 55 predicted CDS, and phages P-PSG-11 and P-PSG-11-1 contain 40,313 bp of DNA with 48 predicted CDS. All phages were double-stranded circular DNA bacteriophages. According to Blastn analysis and phylogenetic analysis of the complete phage genomes (Figure 3), phages Wc4 and Wc4-1 share no significant sequence similarity with other phages. Phages CX5 and CX5-1 showed 93.55% and 93.54% identity with *Pectobacterium* phage PP16 (GenBank accession no. NC_031068.2), respectively, which is the most similar phage (E-value, 0.0; query coverage, 83%). Phages P-PSG-11 and P-PSG-11-1 have 94.86% identity with *Ralstonia* phage RsoP1EGY (GenBank accession no. MG711516.1), which is the most similar phage (E-value, 0.0; query coverage, 88%) and was isolated in Egypt [48]. No studies have been made on the adaptability of these phages to temperature, however. It is also shown that Wc4 and CX5 belong to different clades of *Pectobacterium* phages in the phylogenetic tree (Figure 3A). The genomic sequences were also used to predict the morphology of the phages. Wc4 was found to belong to the *Myoviridae* family, while CX5 and P-PSG-11 belong to the *Podoviridae* family. This was confirmed by transmission electron microscopy (Figure 4).

### 3.7. Genomic Variation in the Ancestral Phages and Adapted Phages 

Comparative genomic results showed that some mutations (substitutions) occurred in the coding region of the adapted phages genomes (Table 2), and no insertions or deletions were found. No mutations occurred in non-coding regions. The mutated gene of the adapted phages Wc4-1 encodes a putative protein with unknown function. Interestingly, the mutations of phage CX5-1 and P-PSG-11-1 occurred on tail tubular proteins gp12 and gp11, respectively. 

### 3.8. Stability of the Mutations during Growth under Non-Selective Conditions

NGS (Next-Generation Sequencing) results showed that the coverage was 100% for both the ancestral and the adapted phages. Further analysis of all the reads in the genome sequence data (the coverage of each position was about 2980) showed that although one genome could be obtained by assembling the majority of reads, there were some very minor reads showing other bases at each nucleotide position, as shown in Table 3. For example, at the genome site 56238, the nucleotide of the ancestor phage Wc4 is C (99.78%) but has 0.21% of the reads showing A, which is the nucleotide of the adapted phage Wc4-1. As shown in Table 3, all the nucleotide positions showed a similar polymorphism. These results reveal that the phages were actually a genetic population, although they were picked from a single plaque. Therefore, it can be speculated that the adapted phage may have been present in the population of the ancestor phage, though at a very minor level. In the adapted phages’ genomes, more than 99.6% of the mapped nucleotides were the nucleotides with corresponding mutations, revealing that the mutations are stably maintained within the adapted phage populations even under non-selective conditions. 

### 3.9. Morphology of the Phages

Morphological examination of the three ancestral phages by transmission electron microscopy (Figure 4) showed that Wc4 belongs to the *Myoviridae* family, with an icosahedral capsid (57.5 ± 5.0 nm, *n* = 5) and a long contractile tail (97.3 ± 3.6 nm, *n* = 5). The phages CX5 and P-PSG-11 belong to the family *Podoviridae*, with a short non-contractile tail of 18 ± 2.0 nm and 12.0 ± 2.3 nm of length, respectively. The icosahedral capsid head diameters of phages CX5 and P-PSG-11 are 55.6 ± 3.0 nm and 42.7 ± 2.6 nm, respectively (mean ± SD; *n* = 5).

## 4. Discussion

This study explores the feasibility of improving the long-term stability of phages through adaptive evolution under elevated thermal conditions. The three obtained adapted phages showed a titer reduction between 0.20 ± 0.13 and 0.61 ± 0.18 log10 after 14 days of storage at 37 °C, while the ancestral phages showed titer declines between 0.78 ± 0.17 and 1.41 ± 0.16 log during the same period. These results demonstrate that adaptive evolution in the lab can be used to enhance the thermal stability of phages. However, it is important to note that while the adaptive evolution process improved the stability of the three adapted phages during storage at 37 °C, a decline was still observed. Therefore, the adaptive evolution process may need to be improved to ensure even better phage stability. Although phages may not necessarily be stored at 37 °C, ambient storage conditions are not always constant, and temperatures are likely to fluctuate especially in hot climates where refrigeration is not available. Other factors such as phage type and solute available in a sample may also influence phage stability [49]. 

The phages P-PSG-11 and Wc4 used in this study have previously been shown to be potential biocontrol agents of the potato phytobacteria *R. solanacearum* [31] and *P. carotovorum* subsp. *carotovorum* [32], respectively. Improved stability would be especially beneficial to ensure that the phages can survive the temperature in the plant environment following application. At the same time, improved stability would mean less titer reduction during storage and transportation, which is also needed for ensuring the efficacy of phage therapy. For example, in a recent clinical trial, the reduction in the phage titer during storage might be a possible reason for the failure of the PhagoBurn trial [50].

In this study, the thermal stabilities of all three phages was improved by adaptive evolution in the lab. Therefore, it is possible to use adaptive evolution as a general method of improving the stability of phages for real applications. Furthermore, changes were not observed in the infectivity and lytic activity of the adapted phages on the host strains tested (Supplementary Figures S1A, S2A, S3A). 

Whole-genome analysis revealed that substitutions in the genomic sequences of the tubular proteins gp11 (P-PSG-11-1) and gp12 (CX5-1) were the only type of mutation observed in the adapted phages (Table 2). It is not yet clear how the single mutations in the tubular proteins contributed to the increased thermal tolerance of the adapted phages. One possible explanation is that the mutated amino acid residues of the tubular proteins improve the phage conformation stability under high temperatures, so that the adapted phage can recognize the host and deliver its genome. However, this needs to be confirmed by testing phage absorption at 60 °C. The morphology of the ancestral phages P-PSG-11 and CX5 revealed that these phages are similar to the T7 phage, which also belongs to the *Podoviridae* family and has a short, non-contractile tail. The tail is a complex formed by a central tubular structure that makes up the channel for DNA ejection, which is surrounded by fibers that are essential in the initial steps of host recognition. The tail tubular proteins gp11 and gp12 have been proposed to form the central tubular structure of the tail [51,52]. Previous studies have shown that a conformational change is needed in the tail for genome delivery [53]. Normally, the tail of *Podoviridae* phages formed of tubular proteins and fibers is involved in host recognition and genome delivery [54].

Lastly, we found that some phages remained active after the first round of heat treatment at 60 °C, although most of the phages were deactivated. These results suggest that the ancestral phage population would contain some heat-resistant mutants. The genome sequencing results also showed that there is polymorphism at each mutant position of the ancestral and adapted phages’ genomes (Table 3). Consecutive cycles of heat treatment and amplification gradually enriched the genetically stable heat-resistant mutations. After five cycles, phages with higher heat resistance would become dominant in the adapted phage population. However, it is also highly possible that the genotype compositions after heat treatments 1, 2, 3, 4, and 5 could be quite different from those of the sequenced phages amplified from single plaques, and the thermo-tolerant phenotypes observed after the fifth heat treatment could be due to different mutants of the adapted phages. Further studies, such as next-generation sequencing, characterizing the phage population after the fifth treatment instead of phages from single plaques, are required to confirm or deny these conclusions. Therefore, it is not surprising to find one mutation per genome after five generations of a heat-treated population, because it likely is the most prevalent genotype in the adapted phage population. 

## 5. Conclusions

To conclude, phage evolution at elevated thermal conditions was found able to improve the stability of phages during storage, without affecting the infectivity and the lytic efficacy of the phages. Single substitutions in the genomic sequences of tubular proteins were observed in the adapted phages. To our knowledge, there is little information on stabilizing phage suspensions, especially phytobacterial phages, via natural selection. Evolving phages to adapt them to high thermal conditions could enable longer storage and easier transportation of the phages, with better activities for real applications. These results might also help to understand the ability of phages to adapt to rising environmental temperatures due to the process of global warming. 

## Figures and Tables

**Table 1:** Information on the bacterial hosts and phages used.

Bacteria	Phage	Source	Area and Year of Phage Isolation
*Pectobacterium carotovorum* subsp *carotovorum* (Pcc) strain KPM17	Wc4 [32]	Soil	Wuhan, China (2016)
*Pectobacterium atrosepticum* (Pba) strain WHG10001	CX5	Fresh Water	Wuhan, China (2017)
*Ralstonia solanacearum* strain GIM1.74.	P-PSG-11 [31]	Fresh Water	Migori, Kenya (2014)

**Table 2:** Mutations observed in the adapted phages.

Adapted Phage	Genome Size (bp)	Genome Position	Protein Encoded by the Mutant Gene	Protein Length (aa)	Amino Acid Change and Position
Wc4-1	92,039	56238	Putative protein	261	Asp → Tyr (96)
CX5-1	43,885	17228	Tail tubular protein (gp12)	776	Tyr → Cys (555)
P-PSG-11-1	40,313	7037	Tail tubular protein (gp11)	195	Val → Ala (81)

**Table 3:** Percentages of the nucleotides mapped to the positions on the phage genomes.

Phage	Nucleotide	Position in Genome	Percentage or Average Percentage of Reads Mapped to the Nucleotides (% of All Reads)			
			A	T	C	G
Wc4	C	56238	0.21	0	99.78	0.01
		All positions	0.29	0.01	99.68	0.02
Wc4-1	A	56238	99.61	0.05	0.33	0.01
		All positions	99.69	0.05	0.25	0.01
CX5	A	17228	98.61	0.15	0.93	0.31
		All positions	99.71	0.10	0.18	0.01
CX5-1	G	17228	0.01	0.15	0.02	99.82
		All positions	0.01	0.16	0.02	99.81
P-PSG-11	T	7037	0.13	99.74	0.01	0.12
		All positions	0.09	99.77	0.01	0.13
P-PSG-11-1	C	7037	0.12	0.01	99.87	0
		All positions	0.14	0.01	99.83	0.02

## Supplementary Materials

The following are available online at https://www.mdpi.com/1999-4915/12/4/423/s1: Supplementary Figures S1–S3: Comparison of the lytic curves of the ancestral and adapted phages and growth curves of the host bacteria. Supplementary Tables S1 and S2: Bacterial strains used in this study.

## References

[1] Suttle C.A. (2005). Viruses in the sea. Nature.

[2] Hatfull G.F. (2015). Dark matter of the biosphere: The amazing world of bacteriophage diversity. J. Virol..

[3] Navarro F., Muniesa M. (2017). Phages in the human body. Front. Microbiol..

[4] Letarov A., Kulikov E. (2009). The bacteriophages in human- and animal body-associated microbial communities. J. Appl. Microbiol..

[5] Muniesa M., Imamovic L., Jofre J. (2011). Bacteriophages and genetic mobilization in sewage and faecally polluted environments. Microb. Biotechnol..

[6] Suttle C.A. (2007). Marine viruses — major players in the global ecosystem. Nat. Rev. Microbiol..

[7] Ashelford K.E., Day M.J., Fry J.C. (2003). Elevated abundance of bacteriophage infecting bacteria in soil. Appl. Environ. Microbiol..

[8] Prigent M., Leroy M., Confalonieri F., Dutertre M., DuBow M.S. (2005). A diversity of bacteriophage forms and genomes can be isolated from the surface sands of the sahara desert. Extremophiles.

[9] Prestel E., Salamitou S., DuBow M.S. (2008). An examination of the bacteriophages and bacteria of the namib desert. J. Microbiol.

[10] Zablocki O., Adriaenssens E.M., Cowan D. (2016). Diversity and ecology of viruses in hyperarid desert soils. Appl. Environ. Microbiol..

[11] Kakasis A., Panitsa G. (2019). Bacteriophage therapy as an alternative treatment for human infections. A comprehensive review. Int. J. Antimicrob. Agents.

[12] Svircev A., Roach D., Castle A. (2018). Framing the future with bacteriophages in agriculture. Viruses.

[13] LeLièvre V., Besnard A., Schlusselhuber M., Desmasures N., Dalmasso M. (2019). Phages for biocontrol in foods: What opportunities for salmonella sp. Control along the dairy food chain?. Food Microbiol..

[14] D’Accolti M., Soffritti I., Piffanelli M., Bisi M., Mazzacane S., Caselli E. (2018). Efficient removal of hospital pathogens from hard surfaces by a combined use of bacteriophages and probiotics: Potential as sanitizing agents. Infect. Drug Resist..

[15] Ryan E.M., Gorman S.P., Donnelly R.F., Gilmore B.F. (2011). Recent advances in bacteriophage therapy: How delivery routes, formulation, concentration and timing influence the success of phage therapy. J. Pharm Pharm..

[16] Vandenheuvel D., Lavigne R., Brussow H. (2015). Bacteriophage therapy: Advances in formulation strategies and human clinical trials. Annu. Rev. Virol..

[17] Zhang Y., Peng X., Zhang H., Watts A.B., Ghosh D. (2018). Manufacturing and ambient stability of shelf freeze dried bacteriophage powder formulations. Int. J. Pharm..

[18] Chang R.Y., Wong J., Mathai A., Morales S., Kutter E., Britton W., Li J., Chan H.K. (2017). Production of highly stable spray dried phage formulations for treatment of pseudomonas aeruginosa lung infection. Eur. J. Pharm. Biopharm..

[19] Merabishvili M., Vervaet C., Pirnay J.P., De Vos D., Verbeken G., Mast J., Chanishvili N., Vaneechoutte M. (2013). Stability of staphylococcus aureus phage isp after freeze-drying (lyophilization). PLoS ONE.

[20] Leung S.S.Y., Parumasivam T., Nguyen A., Gengenbach T., Carter E.A., Carrigy N.B., Wang H., Vehring R., Finlay W.H., Morales S. (2018). Effect of storage temperature on the stability of spray dried bacteriophage powders. Eur. J. Pharm. Biopharm..

[21] Leung S.S., Parumasivam T., Gao F.G., Carrigy N.B., Vehring R., Finlay W.H., Morales S., Britton W.J., Kutter E., Chan H.K. (2016). Production of inhalation phage powders using spray freeze drying and spray drying techniques for treatment of respiratory infections. Pharm. Res..

[22] Jonczyk E., Klak M., Miedzybrodzki R., Gorski A. (2011). The influence of external factors on bacteriophages--review. Folia Microbiol. (Praha).

[23] Lázaro E., Arribas M., Cabanillas L., Román I., Acosta E. (2018). Evolutionary adaptation of an rna bacteriophage to the simultaneous increase in the within-host and extracellular temperatures. Sci. Rep..

[24] Kashiwagi A., Kadoya T., Kumasaka N., Kumagai T., Tsushima F.S., Yomo T. (2018). Influence of adaptive mutations, from thermal adaptation experiments, on the infection cycle of rna bacteriophage qbeta. Arch. Virol..

[25] Kashiwagi A., Sugawara R., Sano Tsushima F., Kumagai T., Yomo T. (2014). Contribution of silent mutations to thermal adaptation of rna bacteriophage qbeta. J. Virol..

[26] Cox J., Schubert A.M., Travisano M., Putonti C. (2010). Adaptive evolution and inherent tolerance to extreme thermal environments. BMC Evol. Biol..

[27] Holder K.K., Bull J.J. (2001). Profiles of adaptation in two similar viruses. Genetics.

[28] Tom E.F., Molineux I.J., Paff M.L., Bull J.J. (2018). Experimental evolution of uv resistance in a phage. Peer J..

[29] Mansfield J., Genin S., Magori S., Citovsky V., Sriariyanum M., Ronald P., Dow M., Verdier V., Beer S.V., Machado M.A. (2012). Top 10 plant pathogenic bacteria in molecular plant pathology. Mol. Plant Pathol..

[30] Czajkowski R., Pérombelon M., Jafra S., Lojkowska E., Potrykus M., van der Wolf J., Sledz W. (2015). Detection, identification and differentiation of pectobacterium and dickeya species causing potato blackleg and tuber soft rot: A review. Ann. Appl. Biol..

[31] Wei C., Liu J., Maina A.N., Mwaura F.B., Yu J., Yan C., Zhang R., Wei H. (2017). Developing a bacteriophage cocktail for biocontrol of potato bacterial wilt. Virol. Sin..

[32] Muturi P., Yu J., Maina A.N., Kariuki S., Mwaura F.B., Wei H. (2019). Bacteriophages isolated in china for the control of pectobacterium carotovorum causing potato soft rot in kenya. Virol. Sin..

[33] Buttimer C., Hendrix H., Lucid A., Neve H., Noben J.P., Franz C., O’Mahony J., Lavigne R., Coffey A. (2018). Novel n4-like bacteriophages of *pectobacterium atrosepticum*. Pharmaceuticals.

[34] Sambrook J., Fritsch E.F., Maniatis T. (1989). Molecular Cloning: A Laboratory Manual.

[35] Bolger A.M., Lohse M., Usadel B. (2014). Trimmomatic: A flexible trimmer for illumina sequence data. Bioinformatics.

[36] Bankevich A., Nurk S., Antipov D., Gurevich A.A., Dvorkin M., Kulikov A.S., Lesin V.M., Nikolenko S.I., Pham S., Prjibelski A.D. (2012). Spades: A new genome assembly algorithm and its applications to single-cell sequencing. J. Comput. Biol..

[37] Li D., Liu C.M., Luo R., Sadakane K., Lam T.W. (2015). Megahit: An ultra-fast single-node solution for large and complex metagenomics assembly via succinct de bruijn graph. Bioinformatics.

[38] Altschul S.F., Gish W., Miller W., Myers E.W., Lipman D.J. (1990). Basic local alignment search tool. J. Mol. Biol..

[39] Katoh K., Standley D.M. (2013). Mafft multiple sequence alignment software version 7: Improvements in performance and usability. Mol. Biol. Evol..

[40] Price M.N., Dehal P.S., Arkin A.P. (2009). Fasttree: Computing large minimum evolution trees with profiles instead of a distance matrix. Mol. Biol. Evol..

[41] Seemann T. (2014). Prokka: Rapid prokaryotic genome annotation. Bioinformatics.

[42] Hyatt D., Chen G.L., Locascio P.F., Land M.L., Larimer F.W., Hauser L.J. (2010). Prodigal: Prokaryotic gene recognition and translation initiation site identification. BMC Bioinform..

[43] Delcher A.L., Kasif S., Fleischmann R.D., Peterson J., White O., Salzberg S.L. (1999). Alignment of whole genomes. Nucleic Acids Res..

[44] Li H., Durbin R. (2009). Fast and accurate short read alignment with burrows-wheeler transform. Bioinformatics.

[45] Li H., Handsaker B., Wysoker A., Fennell T., Ruan J., Homer N., Marth G., Abecasis G., Durbin R., Genome Project Data Processing S. (2009). The sequence alignment/map format and samtools. Bioinformatics.

[46] Kumar S., Stecher G., Tamura K. (2016). Mega7: Molecular evolutionary genetics analysis version 7.0 for bigger datasets. Mol. Biol. Evol..

[47] Lopes A., Tavares P., Petit M.A., Guerois R., Zinn-Justin S. (2014). Automated classification of tailed bacteriophages according to their neck organization. BMC Genom..

[48] Ahmad A.A., Elhalag K.M., Addy H.S., Nasr-Eldin M.A., Hussien A.S., Huang Q. (2018). Sequencing, genome analysis and host range of a novel ralstonia phage, rsop1egy, isolated in egypt. Arch. Virol..

[49] González-Menéndez E., Fernández L., Gutiérrez D., Rodríguez A., Martínez B., García P. (2018). Comparative analysis of different preservation techniques for the storage of staphylococcus phages aimed for the industrial development of phage-based antimicrobial products. PLoS ONE.

[50] Jault P., Leclerc T., Jennes S., Pirnay J.P., Que Y.A., Resch G., Rousseau A.F., Ravat F., Carsin H., Le Floch R. (2019). Efficacy and tolerability of a cocktail of bacteriophages to treat burn wounds infected by pseudomonas aeruginosa (phagoburn): A randomised, controlled, double-blind phase 1/2 trial. Lancet Infect. Dis..

[51] Gonzalez-Garcia V.A., Bocanegra R., Pulido-Cid M., Martin-Benito J., Cuervo A., Carrascosa J.L. (2015). Characterization of the initial steps in the t7 DNA ejection process. Bacteriophage.

[52] Kemp P., Garcia L.R., Molineux I.J. (2005). Changes in bacteriophage t7 virion structure at the initiation of infection. Virology.

[53] Gonzalez-Garcia V.A., Pulido-Cid M., Garcia-Doval C., Bocanegra R., van Raaij M.J., Martin-Benito J., Cuervo A., Carrascosa J.L. (2015). Conformational changes leading to t7 DNA delivery upon interaction with the bacterial receptor. J. Biol. Chem..

[54] Cuervo A., Chagoyen M., Pulido-Cid M., Camacho A., Carrascosa J.L. (2013). Structural characterization of t7 tail machinery reveals a conserved tubular structure among other podoviridae family members and suggests a common mechanism for DNA delivery. Bacteriophage.

