# Supplementary material for: Application of Adaptive Evolution to Improve the Stability of Bacteriophages during Storage
Source: Viruses. 2020 Apr 9;12(4):423. doi: 10.3390/v12040423 (PMC7232334; doi:10.3390/v12040423)
Supplement: Supplementary file 1 [file viruses-12-00423-s001.pdf]

**Supplementary Information**

Comparison of the Lytic Curves of the wild-type ancestral and adapted phages and growth curves of the host bacteria.

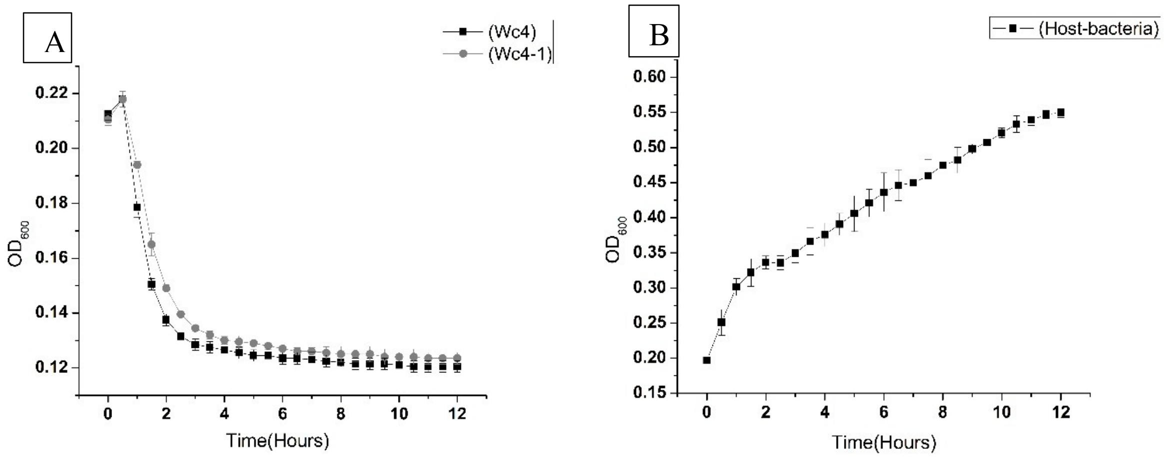


**Figure S1**: (A) Lytic curves of Wc4 and Wc4-1. (B) Growth curve of the host bacterium.

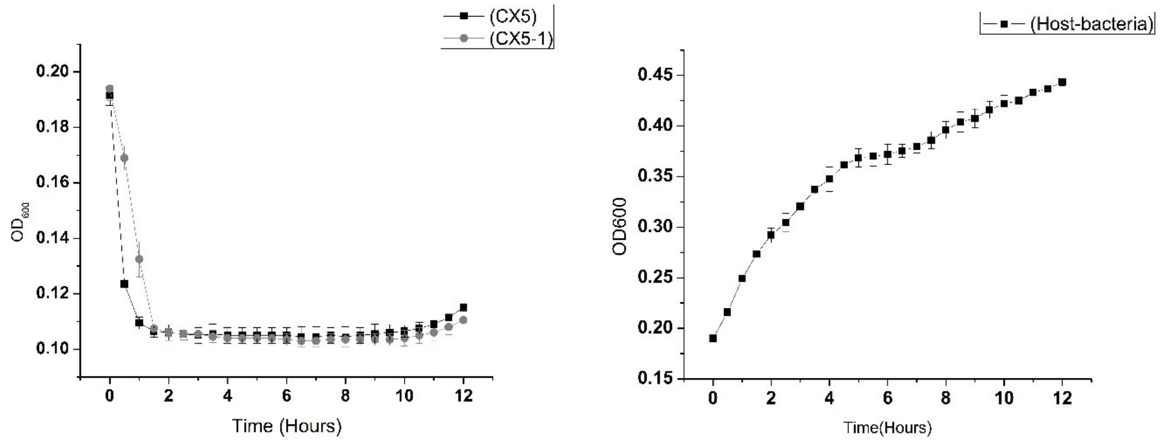


**Figure S2**: (A) Lytic curves of CX5 and CX5-1. (B) Growth curve of the host bacterium.

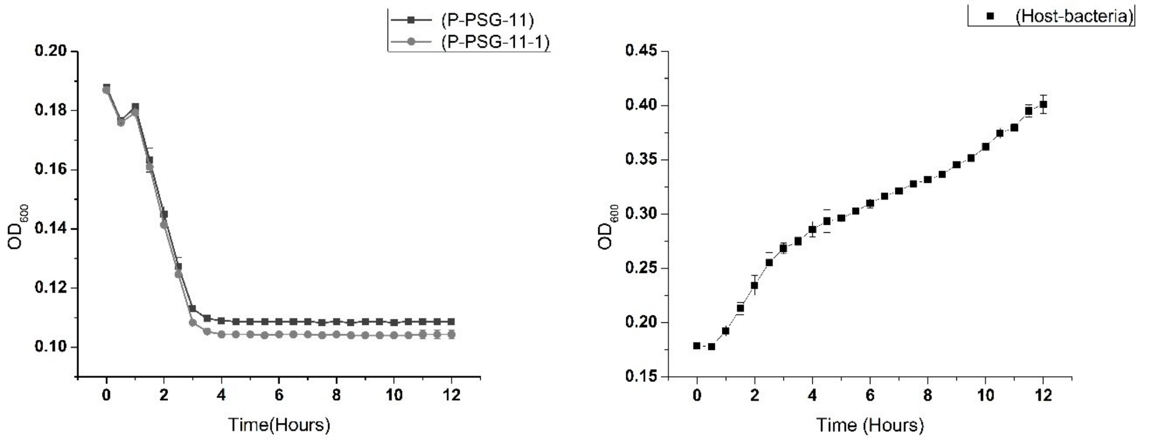


**Figure S3**: (A) Lytic curves of P-PSG-11 and P-PSG-11-1. (B) Growth curve of the host bacterium.

**Table S1**: *Pectobacterium carotovorum* strains used to determine host range of the phages

| Bacteria | Strain | Lytic activity of Wc4 and Wc4-1 |
|---|---|---|
| *Pectobacterium carotovorum* subsp *carotovorum* | KPM13 | ＋+ |
| | KPM14 | ＋+ |
| | KPM17 | ＋+ |
| | KPM30 | ＋+ |
| | KPM32 | ＋+ |
| | KPM51 | ＋+ |
| | KPM58 | ＋+ |
| | KPM59 | ＋+ |
| | KPM62 | ＋+ |
| | KPM76 | ＋+ |
| | KPM74 | ＋+ |
| | KPM84 | ＋+ |
| | KPM88 | ＋+ |
| | KPM94 | ＋+ |
| | KPM99 | ＋+ |
| | KPM102 | ＋+ |

**Table S2**: *Ralstonia solanacearum* and *Pectobacterium atrosepticum* strains used to determine the host range of the phages

| Bacteria | Strain | Lytic activity of P-PSG-11 & P-PSG-11-1 |
|---|---|---|
| *Ralstonia solanacearum* | PS-X4-1 | ＋+ |
| | PS-X10-2 | ＋+ |
| | PS-X13-1 | ＋+ |
| | | Lytic activity of CX5 & CX5-1 |
| *Pectobacterium atrosepticum* | WHG10001 | ＋+ |

The phages were lytic (＋; clear spot, +; plaques formed) to the bacterial strains tested. The relative number of plaques generated by either the adapted or ancestral phages was about the same for the bacterial strains tested. Efficiency of plating (EOP) was almost 100 % for each adapted phage versus its respective wild-type ancestor phage.
